# *Philadelphus tenuifolius* Leaf Extract Exhibits Anti-Tuberculosis Activity by Enhancing Host Autophagy and Immunity: A Promising Host-Directed Therapeutic Candidate

**DOI:** 10.4014/jmb.2601.01032

**Published:** 2026-03-26

**Authors:** Tam Doan Nguyen, Ji-Ae Choi, Doyi Son, Heung Joo Yuk, Junghwan Lee, Sanghun Son, Jaewhan Kim, SeoYeon Jo, Dong-Seon Kim, Chang-Hwa Song

**Affiliations:** 1 Department of Microbiology, College of Medicine, Chungnam National University, Daejeon 35015, Republic of Korea; 2 Department of Medical Science, College of Medicine, Chungnam National University, Daejeon 35015, Republic of Korea; 3 Translational Immunology Institute, Chungnam National University, Daejeon 34134, Republic of Korea; 4 Herbal Medicine Research Division, Korea Institute of Oriental Medicine, Daejeon 34054, Republic of Korea

**Keywords:** Tuberculosis, Host-directed therapy, *Philadelphus tenuifolius*, MAPK signaling, Autophagy, Natural products

## Abstract

The global emergence of drug-resistant *Mycobacterium tuberculosis* (Mtb) necessitates the urgent discovery of novel anti-tuberculosis agents. *Philadelphus tenuifolius* Rupr., a Korean aromatic herb, has been historically recognized for its traditional medicinal uses. This study aimed to scientifically investigate the anti-tuberculosis (TB) potential of the *P. tenuifolius* leaf extract (PT-LE) and elucidate its underlying mechanism of action. PT-LE was prepared by 50% ethanolic extraction. Its anti-TB activity was evaluated against intracellular Mtb in BMDMs. Mechanistic studies focused on the activation of the MAPK pathway and autophagy flux. Synergistic effects with conventional anti-TB drugs were also assessed. For the *in vivo* evaluation, Mtb-infected mice were orally treated daily with PT-LE (100 mg/kg), followed by the determination of the lung bacterial burden. PT-LE exhibits negligible host cell cytotoxicity and effectively reduced the bacterial load of intracellular Mtb within macrophages. Mechanistically, PT-LE was shown to significantly activate the MAPK signaling pathway, which subsequently enhanced autophagy flux – a critical host defense mechanism against Mtb. Furthermore, PT-LE demonstrated potent synergistic activity with existing anti-TB drugs and modulated the host immune response by increasing the production of the chemokine MCP-1. Critically, the *in vivo* experiments showed that oral administration of PT-LE significantly reduced the Mtb bacterial burden in the lungs of infected mice. These findings reveal a novel anti-TB function of PT-LE, which operates by enhancing host autophagy and immunity. PT-LE represents a promising and effective host-directed therapeutic candidate for both drug-sensitive and drug-resistant tuberculosis.

## Introduction

Tuberculosis (TB) remains a major global health challenge, further exacerbated by the emergence of multidrug-resistant (MDR) and extensively drug-resistant (XDR) strains of *Mycobacterium tuberculosis* (Mtb). TB drug-resistant strains require longer therapy, often leading to poor patient compliance and serious side effects, thereby significantly reducing the overall effectiveness of existing chemotherapy options [1, 2]. Recently, a new all-oral BPaL regimen with bedaquiline, pretomanid and linezolid was approved to cure adults with drug-resistant TB in 2019 [3]. Although this regimen is successfully used worldwide, ongoing challenges such as drug toxicity severe side effects, and the potential emergence of resistance highlight the urgent need for additional therapeutic strategies [4].

Considering the current challenges in TB treatment, host-directed therapy (HDT), which bolsters the host’s own immune mechanisms rather than targeting the pathogen directly, is an emerging approach [5, 6]. Several studies have identified FDA approved drugs targeting cell death, anti-inflammatory response, immune-modulation and regulating metabolic pathways that have entered clinical trials for the treatment of TB [7-11]. Furthermore, some repurposed drugs with autophagy-inducing potential, such as phenylbutyrate, vitamin D3, statins, atorvastatin, and pravastatin, have been investigated in Phase II clinical trials as HDT for TB treatment [7, 8].

In recent years, natural products derived from plants have received considerable attention due to their immunomodulatory effects in TB treatment [12, 13]. For instance, flavonoids such as baicalin and baicalein have shown anti-TB effects by promoting autophagy through the suppression of the Akt/mTOR pathway and by inhibiting inflammation through the down-regulation of AIM2 and NLRP3 inflammasomes in Mtb-infected macrophages [14, 15]. The polyphenolic flavonoid, Quercetin (from capers, red onion, kale), which down-regulates the NLRP3 inflammasome, has already entered Phase II clinical trials for TB treatment [7]. Besides, another polyphenolic flavonoid, epigallocatechin gallate (from green tea), which induces autophagy through the enhancement of autolysosome acidification, has been investigated as an HDT candidate in a randomized clinical trial for TB treatment [7, 16].

*Philadelphus schrenkii* complex (Hydrangeaceae) is native to East Asia, including regions of China, Korea, Japan, and Far Eastern Russia [17]. Phytochemical analyses of *Philadelphus* species have revealed the presence of bioactive compounds such as flavonoids and coumarins, which are associated with antioxidant, hepatoprotective, antidiabetic properties, and anti-inflammatory effects [18, 19]. A previous study has shown that the leaf of *P. schrenkii* inhibited the growth of *Pseudomonas aeruginosa*, and the leaf and flower of *P. schrenkii* inhibited the growth of the drug-resistant strain *Staphylococcus aureus* SA2 when treated with chloramphenicol [20]. In addition, another study demonstrated that three different species of *Philadelphus* (*P. microphyllus*, *P. coronarius* and *P. pekinensis*) containing flavonoids and triterpenes have anti-bacterial effects against *P. aeruginosa*, *S. aureus* and *Enterococcus faecalis* comparable to ampicillin [21].

*P. tenuifolius*, a member of the *Philadelphus* genus, has been used traditionally for hemorrhoids (unripe fruits and roots) and valued for its tonic and diuretic effects (flowers) in Korea [22]. It is known that the main component of *P. tenuifolius* leaves is flavonoids, similar to other species [23]. However, the anti-TB efficacy and underlying mechanisms of *P. tenuifolius* have yet to be reported. Therefore, in the present study, we aimed to investigate the ant antimicrobial and host-directed activity of *P. tenuifolius* leaf extracts (PT-LE) against Mtb.

## Materials and Methods

### Reagents

RIF, INH, EMB, and Lipopolysaccharide (LPS) were purchased from Sigma (USA). MAPK inhibitors for ERK (PD98059), JNK (SP600125) and p38 (MT4) were purchased from Calbiochem (USA). These reagents were dissolved in dimethyl sulfoxide (Sigma) or distilled water.

### Plant Material and Extraction

Dried leaves of *Philadelphus tenuifolius* (harvested in Mungyeong, Korea, in August 2024) were purchased from KOCBIO, Inc. (Republic of Korea; http://jcode.inckorea.net). Botanical identity was authenticated by the supplier. One kilogram (1 kg) of dried leaves was extracted with 50% (v/v) ethanol (15 L) under reflux for 3 h. The extract was filtered through a 5 μm cartridge filter, concentrated under reduced pressure using a rotary evaporator (EV-1020, SciLab, Republic of Korea), and subsequently freeze-dried (LP20, IlshinBioBase, Republic of Korea) to obtain a powdered extract. The resulting extract powder was homogenized, vacuum-packed, and stored at –20°C until further use in biological assays (Yield: approx. 33% w/w). Building on this prior phytochemical characterization [24], the present study performed comparative HPLC fingerprinting using the PT-LE employed in all biological experiments and an authenticated standard extract obtained from the Plant Extract Bank at the Korea Research Institute of Bioscience and Biotechnology (KRIBB) to confirm their chemical consistency.

### Cell Cultures

Bone marrow derived macrophages (BMDMs) were isolated from femurs and tibias of C57BL/6 mice (6-9 weeks old, Samtako, Republic of Korea). Cells were differentiated for 4–5 days in Dulbecco’s Modified Eagle Medium, DMEM (Welgene. Republic of Korea) supplemented with macrophage colony-stimulating factor (M-CSF, 25 μg/ml; R&D Systems, USA) at 37°C and 5% CO_2_.

### Bacteria Culture and Infection

*Mycobacterium tuberculosis* H37Rv (Mtb; ATCC 27294) was cultured in Middlebrook 7H9 liquid medium (BD Difco, USA) supplemented with 10% Olec acid-Albumin-Dextrose-Catalase (OADC, BD BBL, USA), 0.2% glycerol, and 0.05% Tween 80. Cultures were grown at 37°C with shaking (140 rpm) until the log phase (OD_600_ = 0.6) was reached. Bacteria were harvested by centrifugation (3000 ×*g* for 30 min), washed three times with PBS, resuspended in PBS, and frozen at –70°C. Thawed aliquots were dispersed briefly using a bath sonicator before use. CFU/ml was determined by serial dilution in 7H9 medium and plating on 7H10 agar.

### Cell Viability Assay

Cell viability was measured using Cell Counting Kit-8 assay (CCK-8; Dojindo, Japan). BMDMs (5 × 10^3^ cells/well) were seeded in 96 well plates and treated with PT-LE (25, 50 and 100 μg/ml). After 24 h incubation, CCK-8 dye (10 μl) was added, and the cells were incubated for another 1 h at 37°C. 10% (v/v) DMSO was used as a positive control. Absorbance was measured at 450 nm.

### *In vitro* Antimicrobial Efficacy: Minimum Inhibitory Concentration (MIC), Minimum Bactericidal Concentration (MBC), Fraction Inhibitory Concentration (FIC)

PT-LE was dissolved in DMSO (20 mg/ml) and subjected to serial dilution (100 to 0.39 μg/ml) in 7H9 broth with 10% OADC. An adjusted bacterial inoculum (10^5^/ml CFU) was added to each well. The MIC endpoint was defined as the lowest concentration of the compound showing no visible bacterial growth after 21 days of incubation. For MBC determination, 50 μL aliquots form wells with no visible growth were seeded on 7H10 agar plates. The MBC was defined as the lowest concentration that killed 99% of the bacterial population after 21 days.

Synergistic effects of PT-LE with RIF, INH, or EMB were assessed by the checkerboard method. The FIC index was calculated using the formula: : FIC = (MIC_A combination_/MIC_A alone_) + (MIC_B combination_/MIC_B alone_) [25]. Synergy was defined as FIC ≤ 0.5, indifference as 0.5 < FIC ≤ 4, and antagonism as FIC > 4.

### Intracellular Survival Analysis

The intracellular survival of H37Rv in BMDMs was quantified by CFU analysis. BMDMs were infected with H37Rv at a MOI of 1 for 3 h. Non-phagocytized bacteria were removed by PBS washing, and cells were further incubated for 24 h in fresh, antibiotic-free medium. Intracellular bacteria were collected by lysing the cells with 1 ml of autoclaved distilled water per well [26]. Lysates were plated on 7H10 agar and incubated at 37°C for 21 days.

### Measurement of Nitric Oxide (NO)

NO levels in the culture supernatant of BMDM cells were analyzed using the Griess reagent (100 µl, Promega, USA) for 10 min at room temperature (RT), and absorbance was measured at 541 nm.

### Autophagy Flux Analysis (mRFP-GFP-LC3 Assay)

BMDM cells plated on coverslips were transfected with the mRFP-GFP-tf-LC3 reporter plasmid using the Lipofectamine 3000 Transfection Kit (Invitrogen). After 48 h, cells were infected with H37Rv and then treated with PT-LE for 48 h. Puncta formation was observed under confocal microscope (Zeiss LSM900). The ratio of autolysosomes (red-only puncta) to autophagosomes (yellow puncta) was quantified using ImageJ software to assess autophagic flux [27].

### Western Blot Analysis

BMDMs were treated with PT-LE, infected with H37Rv, or co-treated, and cell lysates were harvested at indicated time points. Proteins were separated by SDS-PAGE and probed with primary antibodies against ERK1/2, p38, JNK, p-ERK1/2, p-p38, p-JNK, p62 and LC3I/II (Cell Signaling Technology, USA).

### Detection of Inflammatory Cytokines

Cell supernatants and lung homogenates were collected and frozen for batch analysis. Proinflammatory cytokines were quantified using a cytometric bead array mouse inflammation kit (BD Biosciences, USA) on a FACS Canto II cytometer (BD Biosciences). Data was processed using Flow Jo (Tree Star).

### *In Vivo* Infection Model and Image Analysis

C57BL/6 female mice (8 weeks of age) were purchased from Samtako Bio Korea (Republic of Korea). Mice were infected intratracheally with Mtb strain H37Rv (10^6^ CFU/50 μl PBS). Two weeks post-infection, mice were orally treated daily with PT-LE (100 mg/kg) or vehicle (0.5% carboxymethylcellulose) for two subsequent weeks (*n* = 7 per group). Lungs were collected 28 days after Mtb infection for CFU and histological analysis. For Hematoxylin and Eosin (H&E) stain, collected lung tissues were fixed in 10% formalin, embedded in paraffin, and sectioned. Tissue sections were stained with Gill’s V Hematoxylin and Eosin Y solution following standard procedures. Stained samples were analyzed with an Axiophot microscope (Carl Zeiss, Germany). To quantify lung tissue pathology, the percentage of the damaged area was calculated using ImageJ software (NIH, USA). All animal procedures were performed under protocols reviewed and approved by the Institutional Animal Care and Use Committee of Chungnam National University, Daejeon, Republic of Korea (permit no. 202401A-CNU-019), and were conducted in accordance with the guidelines of the Korean Food and Drug Administration.

### Statistical Analysis

Statistical analyses were conducted using GraphPad Prism Software (version 8.0; GraphPad Software, USA). Data from independent experiments are presented as means ± SD. All experiments were repeated at least three times. One-way analysis of variance (ANOVA) followed by Dunnett multiple comparisons test between control and experimental groups was employed. Statistical significance was defined as *p < 0.05, **p < 0.01, ***p < 0.001 and ****p < 0.0001.

## Results

### PT-LE Reduces the Intracellular Survival of Mtb in BMDMs

**Fig. 1:**
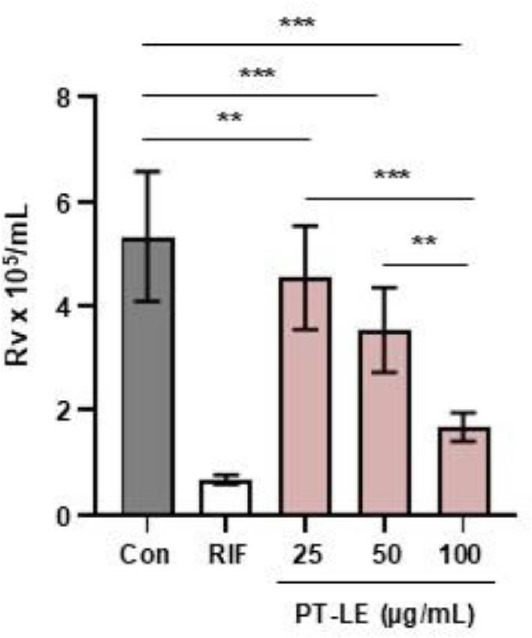
PT-LE significantly decreases the intracellular survival of Mtb. BMDMs were infected with H37Rv (MOI = 1) and then treated with vehicle control (Con), RIF (0.1 μg/ml), or increasing concentration of PT-LE (25, 50, and 100 μg/ml) for 24 h. Intracellular bacterial burden was assessed by CFU enumeration. Data are presented as mean ± SD of triplicate wells and are representative of at least three independent experiments. Statistical significance was determined using one-way ANOVA followed by Dunnett’s multiple comparison test. **p < 0.01, ***p < 0.001. RIF, rifampicin.

In order to determine whether PT-LE can affect Mtb growth, BMDMs were infected with Mtb at a multiplicity of infection (MOI) 1 for 3 h and then stimulated with PT-LE for 24 h. Comparative HPLC fingerprinting confirmed that the PT-LE contained the marker compounds rutin and nicotiflorin (Fig. S1A), identically mirroring the chromatographic profile of an authenticated KRIBB reference extract (Fig. S1B). The initial assessment was conducted to determine the intracellular survival of Mtb in BMDMs following PT-LE stimulation. Intracellular Mtb growth was significantly reduced in BMDMs after PT-LE treatment in a dose-dependent manner compared to the untreated control (Fig. 1). Furthermore, PT-LE at a concentration of 100 μg/ml exhibited inhibitory efficacy comparable to that of RIF. Cell viability assays revealed that concentrations of up to 100 μg/ml didn’t induce significant cytotoxicity in BMDMs, establishing this level as the maximum non-toxic concentration (Fig. S2). Despite the intracellular antimycobacterial activity of PT-LE, PT-LE did not show direct bactericidal killing effects on Mtb (minimum inhibitory concentration (MIC) and minimum bactericidal concentration (MBC) > 100 μg/ml) (Table S1). These results led us to investigate the effect of PT-LE on the regulation of the host immune response against Mtb infection.

### PT-LE Promotes the Intracellular Killing of Mtb via MAPK Signaling Pathways in BMDMs

**Fig. 2:**
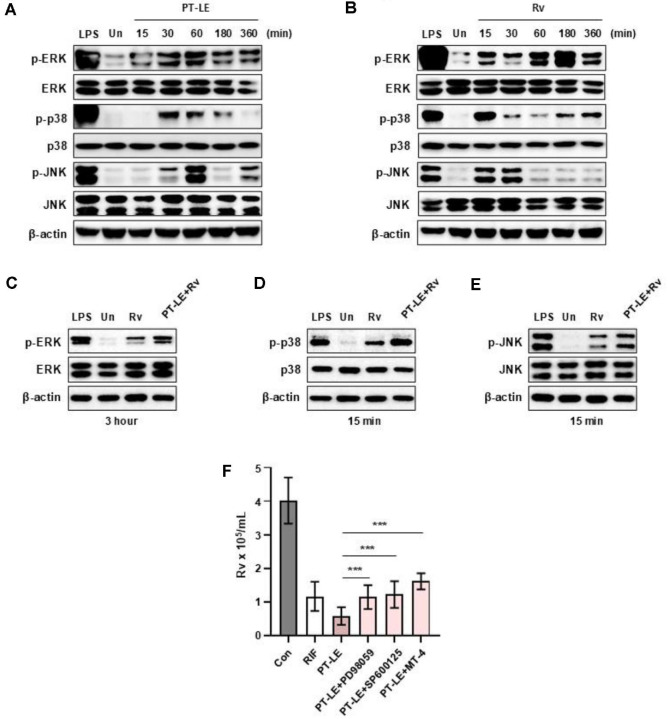
PT-LE enhances MAPK signaling pathways in BMDMs during Mtb infection. (**A, B**) BMDMs were treated with PT-LE (100 μg/ml) (**A**) and infected with Mtb infection (MOI = 1) (**B**) for the indicated times. The phosphorylation levels of ERK1/2, p38, and JNK were analyzed by Western blotting. (**C–E**) BMDMs were infected with Mtb infection (MOI = 1) and then treated with PT-LE (100 μg/ml) for indicated times. The phosphorylation of ERK (**C**), p38 (**D**), and JNK (**E**) was assessed by immunoblotting. LPS (100 ng/ml) was used as a positive control. Total ERK, p38, and JNK, as well as β-actin, were used as loading controls. (**F**) BMDMs were preincubated for 1 hour with specific MAPK pathway inhibitors followed by Mtb (MOI = 1) infection and then treated with PT-LE (100 μg/ml) for 24 h. Intracellular bacterial burden was determined by CFU assay 24 h post-treatment with PT-LE. Data are presented as mean ± SD of triplicates and are representative of at least three independent experiments. Statistical significance was calculated using one-way ANOVA with Dunnett’s post hoc test. ***p < 0.001. Un, uninfected control; RIF, rifampicin.

The MAPK pathway is a well-known crucial host defense mechanism that has been demonstrated to be effective against various pathogens, including Mtb. This pathway has been shown to play a significant role in anti-mycobacterial activity and the production of cytokines following infection [28]. We investigated the potential activation of MAPK signaling pathways in macrophages by PT-LE. The phosphorylation of ERK and p38 peaked between 30 and 60 min, and the level of phospho-JNK reached its peak at 60 min (Fig. 2A). PT-LE induced-MAPK activation was blocked by the specific inhibitors, such as PD98059 (ERK), SP600125 (JNK) and MT4 (p38), respectively (Fig. S3). Furthermore, a parallel observation was made concerning the activation of MAPK following Mtb infection (Fig. 2B). In addition, PT-LE stimulation augmented Mtb-induced MAPK activation in BMDMs (Fig. 2C–2E). Intriguingly, pharmacological inhibition of MAPK signaling significantly abrogated the intracellular Mtb killing effect of PT-LE (Fig. 2F). The results of this study indicate that the PT-LE-activated MAPK pathway plays a critical role in the regulation of Mtb intracellular survival within macrophages.

### The PT-LE Modulates Inflammatory Cytokine Responses to Regulate Mtb Survival in Macrophages

**Fig. 3:**
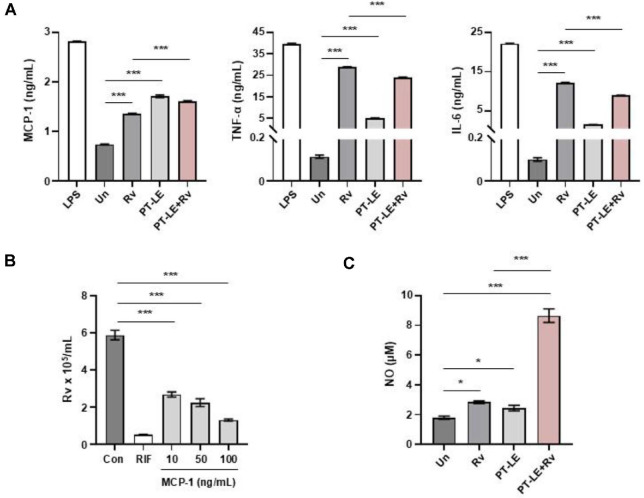
PT-LE regulates inflammatory responses in BMDMs during Mtb infection. (**A**) BMDMs were infected with Mtb infection (MOI = 1) and then treated with PT-LE (100 μg/ml) for 24 h. Cytokine and chemokine production levels of TNF-α, MCP-1 and IL-6 in culture supernatants were measured by ELISA. LPS (100 ng/ml) was used as a positive control. (**B**) BMDMs were infected with H37Rv and then incubated for 24 h in the presence of recombinant MCP-1 (10, 50, or 100 ng/ml) or RIF (0.1 μg/ml). Intracellular bacterial loads were determined by CFU assay. (**C**) BMDMs were infected with Mtb infection (MOI = 1) and then treated with PT-LE (100 μg/ml) for 24 h. NO production was assessed in culture supernatants using the Griess assay. Data are presented as mean ± SD of at least three independent experiments. Statistical significance was determined by one-way ANOVA with Dunnett’s post-hoc test. *p < 0.05 and ***p < 0.001. Un, uninfected control; RIF, rifampicin.

Given the evidence that MAPK pathways in macrophages regulate cytokine production in innate immune responses [28], we investigated whether PT-LE could modulate the inflammatory cytokines production in BMDMs. PT-LE significantly induced the production of inflammatory cytokines including TNF-α, MCP-1, IL-6 and IL-10, in comparison to the control group (Fig. 3A). Specifically, PT-LE treatment led to substantial increase in MCP-1 production in Mtb-infected BMDMs, while Mtb-induced levels of TNF-α and IL-6 were significantly reduced by PT-LE (Fig. 3A). In the following stage of the investigation, the effects of these cytokines on bacterial clearance were examined. Intracellular Mtb survival was assessed in macrophages treated with recombinant MCP-1, TNF-α or IL-6. Among the tested cytokines, only MCP-1 administration resulted in a significant reduction of intracellular Mtb growth in a dose-dependent manner (Fig. 3B), whereas TNF-α and IL-6 had no appreciable effect (Fig. S4). We then proceeded to assess the production nitric oxide (NO) by PT-LE in Mtb-infected BMDMs. As shown in Fig. 3C, PT-LE significantly enhanced NO production was observed during Mtb infection. These findings suggested that the PT-LE-induced MCP-1 and NO are involved in the regulation of intracellular Mtb survival in BMDMs.

### PT-LE Restores Autophagy Flux in Macrophages during Mtb Infection

**Fig. 4:**
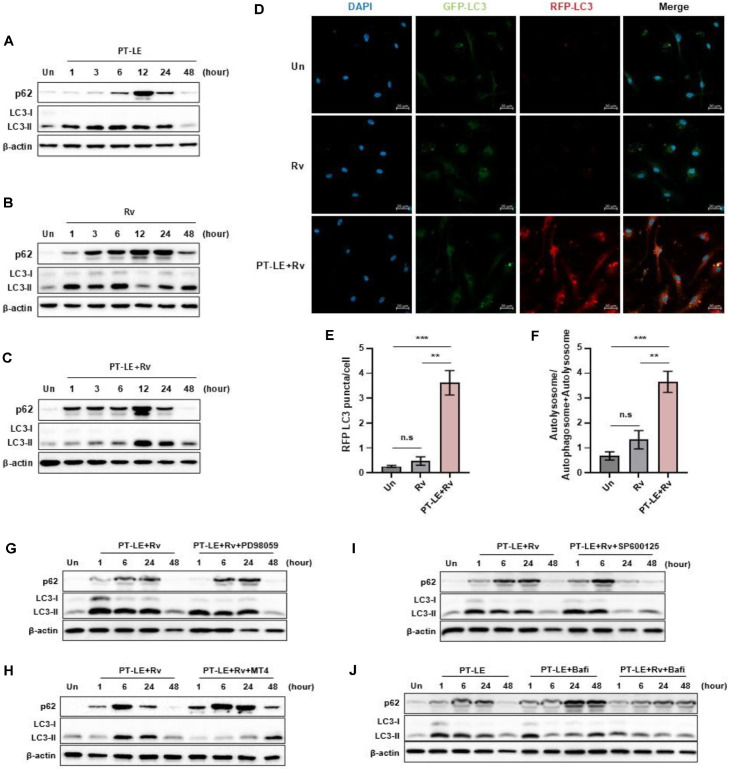
PT-LE significantly induces autophagy in BMDMs during Mtb infection. (**A–C**) Western blot analysis of p62 and LC3 was measured in BMDMs infected with Mtb, treated with PT-LE (100 μg/ml) alone, or treated with PT-LE after Mtb infection (PT-LE+Rv) for the indicated time points. β-actin used as a loading control. (**D-F**) BMDMs were transfected with the mRFP-GFP tandem fluorescent-tagged LC3 (tfLC3) reporter plasmid for 48 h and then treated with PT-LE for 48 h after Mtb infection. Confocal microscopy images showed autophagosome (GFP-LC3) and autolysosome (RFP-LC3) formation in. Nuclei were counterstained with DAPI. Quantification of RFP-LC3 puncta per cell (**E**) and the ratio of autolysosomes to total autophagic vesicles (autophagosomes + autolysosomes) (**F**) were determined from reporter assays. (**G-I**) Western blot analysis of p62 and LC3 in cells pre-treated for 1 hour with inhibitors for ERK (PD98059) (**G**), p38 (MT4) (**H**), or JNK (SP600125) (**I**) prior to PT-LE treatment and Mtb infection. (**J**) Western blot of p62 and LC3 in cells treated with PT-LE or PT-LE+Rv, with or without the autophagy inhibitor Bafilomycin A1 (Bafi). Data represent mean ± SD from at least three independent experiments. Statistical significance was calculated using one-way ANOVA with Dunnett’s post-hoc test. **p < 0.01, ***p < 0.001, n.s, not significant. Un, uninfected control.

To assess the effect of PT-LE on autophagy induction, we measured microtubule-associated protein 1A/1B-light chain 3 (LC3) and p62/SQSTM1 levels in Mtb-infected BMDMs after PT-LE treatment. As indicated by the data presented in Fig. 4A, PT-LE exposure led to an elevation in the levels of p62 and LC3-II for up to 12 h, followed by a subsequent decrease to the baseline level at 48 h. This observation suggests that the treatment of PT-LE resulted in the completion of the autophagic flux. In contrast, Mtb infection induced sustained elevations in the levels of p62 and LC3-II over a period of 48 h. This finding suggests that Mtb infection impedes autophagy flux (Fig. 4B). Notably, PT-LE treatment after Mtb infection resulted in a marked reduction of p62 and LC3-II at 48 h, indicating that PT-LE restored Mtb-mediated autophagy blockade (Fig. 4C). To analyze autophagy dynamics, BMDMs were transduced with the mRFP-GFP tandem fluorescent-tagged LC3 (tfLC3) reporter plasmid and stimulated with PT-LE after Mtb infection. Mtb infection slightly increased the formation of green and red puncta. However, there was no significant increase in the number of autolysosomes (red puncta) or the ratio of autolysosome to autophagosomes (green puncta) compared to controls. These results indicate an impaired autophagic flux in Mtb-infected BMDMs. In contrast, the administration of PT-LE following Mtb infection resulted in a substantial augmentation in the formation of RFP-LC3 puncta and the ratio of autolysosomes to total autophagic vesicles. This observation suggests an enhancement in the maturation of autophagosomes into autolysosomes (Fig. 4D-4F). To determine whether MAPK signaling mediates this process, we pre-treated cells with specific MAPK inhibitors. Inhibition of ERK (PD98059) or JNK (SP600125) did not prevent PT-LE-induced p62 degradation (Fig. 4G and 4I). In contrast, the p38 inhibitor MT4 significantly suppressed both p62 clearance and LC3-II induction, suggesting that p38 MAPK activation is indispensable for PT-LE-mediated autophagy (Fig. 4H). Finally, co-treatment with Bafilomycin A1 (Bafi) caused both p62 and LC3-II to accumulate at 48 h, confirming that PT-LE stimulates complete autophagic flux (Fig. 4J). Collectively, these results demonstrate that PT-LE improved impaired autophagic flux by enhancing the maturation of autophagosomes into autolysosomes in Mtb-infected BMDMs.

### PT-LE Treatment Suppresses the Intracellular Growth of Mtb *In vivo*

**Fig. 5:**
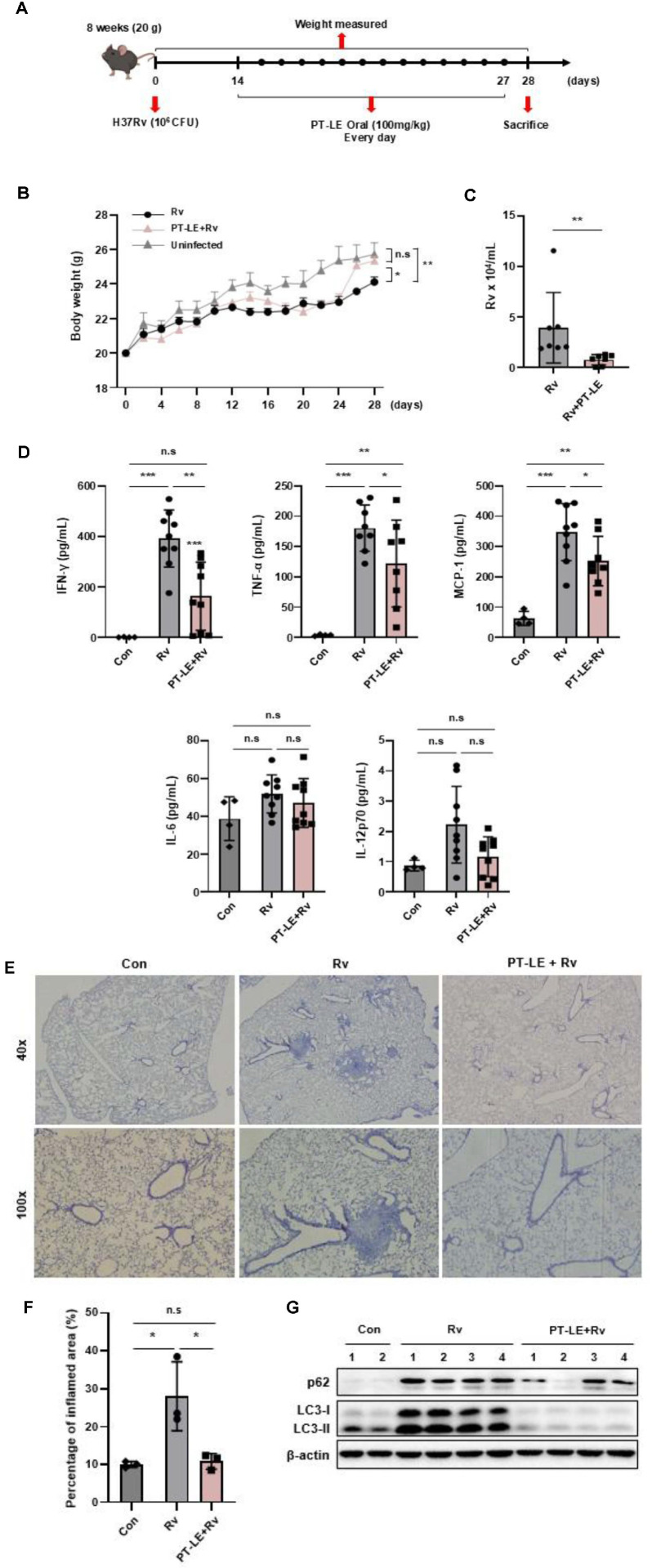
PT-LE inhibits intracellular survival of Mtb *in vivo*. (**A**) Schematic diagram of the experimental time course for PT-LE treatment in the Mtb infection mouse model. C57BL/6 mice were intratracheally infected with Mtb (10^6^ CFU) and then orally administered with PT-LE (100 mg/kg) daily from day 14 to day 28 (sacrifice day). (**B**) Body weight was monitored every 2 days throughout the experiment. (**C**) Bacterial burden in the lungs was determined by CFU assay on day 28. (**D**) Cytokine and chemokine levels in lung homogenates were measured by CBA assay. (**E**) Representative H&E staining of lung tissues showing pathological changes at 40× and 100× magnification. (**F**) Quantitative analysis of the inflamed area percentage in the lung tissue sections. (**G**) Western blot analysis of p62 and LC3, in lung tissue. β-actin used as loading control. Data are presented as mean ± SD of at least three independent experiments. Statistical significance was assessed by one-way ANOVA with Dunnett’s post-hoc test. *p < 0.05, **p < 0.01, ***p < 0.001; n.s, not significant.

In order to evaluate the *in vivo* antimycobacterial efficacy of PT-LE, mice were infected by intratracheal instillation with 10^6^ colony-forming units (CFU) of Mtb. From day 14 to day 27 after infection, mice were orally treated with PT-LE (100 mg/kg/day) once a day (Fig. 5A). A body weight analysis revealed that Mtb-infected mice exhibited a significantly greater loss of weight compared to the control mice. In contrast, from 10 days after receiving PT-LE, the mice exhibited body weights that were not significantly different from those of the control groups. Although an initial decrease in body weight was observed across infected groups due to the physical stress of the intratracheal instillation procedure, by 28 days post-infection, there was no significant difference in body weight between the PT-LE-treated group and the uninfected controls. This complete weight recovery demonstrates that the oral administration of PT-LE effectively prevents Mtb-induced cachexia and is systemically well-tolerated at 100 mg/kg *in vivo* (Fig. 5B). Notably, the oral administration of PT-LE significantly decreased the pulmonary bacterial burden at 4 weeks post-infection, achieving an approximate 0.7 log_10_ reduction in CFU compared to the vehicle-treated infected control (Fig. 5C). In addition, our findings indicate that PT-LE treatment significantly mitigates lung inflammation following Mtb infection. The administration of PT-LE resulted in a significant decrease in the levels of Mtb-induced inflammatory cytokines (IFN-γ, TNF-α and MCP-1) in the lung homogenates. However, there were no significant alterations observed in the levels of IL-6 or IL-12p70 (Fig. 5D). Furthermore, histological analysis of the Mtb-infected lungs revealed severe pathological changes, characterized by dense lymphocytic infiltration, granuloma formation, and widespread alveolar space consolidation (Fig. 5E). Quantitative analysis revealed that oral administration of PT-LE significantly attenuated Mtb-induced immunopathology, reducing the inflammation to levels comparable to the uninfected control (Fig. 5F). Additionally, we observed that levels of p62 and LC3-II were significantly increased in the lungs of the Mtb-infected group compared with the control group. It is important to note that Mtb-induced p62 and LC3-II expression were significantly reduced by PT-LE treatment in the lungs. This suggests that PT-LE treatment has the capacity to restore impaired autophagy flux by Mtb infection *in vivo* (Fig. 5G). Taken together, these results suggest that PT-LE administration suppresses Mtb growth *in vivo* by modulating host inflammatory responses and autophagy activation.

### PT-LE Exhibits Synergistic Antimicrobial Activity with First-Line Anti-TB Drugs against Intracellular Mtb

To explore the potential synergistic effects of PT-LE in combination with anti-TB drugs against intracellular Mtb, we found that the intracellular MIC of PT-LE decreased from 50 μg/ml to 6.25 μg/ml when combined with three anti-TB drugs, compared to treatment with the drugs alone (Table 1). Similarly, the intracellular MICs of Rifampicin (RIF), isoniazid (INH), and ethambutol (EMB) decreased when combined with PT-LE compared to drug treatment alone against intracellular Mtb. Interestingly, strong synergy was observed between PT-LE and the three anti-TB drugs (RIF, INH and EMB) against intracellular Mtb, as indicated by a fractional inhibitory concentration (FIC) of ≤0.5. Combination treatment with EMB (0.3 μg/ml) and PT-LE (6.25 μg/ml) exhibited the most significant synergy (FIC index = 0.2) against intracellular Mtb. Our results indicate that PT-LE exhibits synergistic antimicrobial activity when used in combination with first-line anti-TB drugs. This supports the potential role of PT-LE as a host-directed adjunct therapy to improve the efficacy of current TB treatment regimens.

## Discussion

Chemical standardization is a critical consideration in studies investigating the host-directed therapeutic effects of plant-derived extracts. Previous phytochemical analyses of *Philadelphus tenuifolius* leaf extracts have identified rutin and nicotiflorin as major constituents [24]. Consistent with these reports, our comparative HPLC fingerprinting confirmed the presence of these marker compounds in the PT-LE utilized for all *in vitro* and *in vivo* experiments. This profiling was validated against an authenticated reference extract obtained from the Plant Extract Bank at the Korea Research Institute of Bioscience and Biotechnology (KRIBB). Establishing the chemical consistency of our PT-LE bolsters the reproducibility and translational relevance of the observed host-directed anti-tuberculosis effects.

Natural plant-derived compounds have attracted significant attention for their potential in the development of novel TB treatments due to their multitargeted mechanisms, favorable safety profiles, and cost-effective production [13, 29] In the present study, it was observed that MAPK signaling activation was enhanced by administering PT-LE after Mtb infection. Furthermore, pharmacological inhibition of the MAPK pathway prevented the intracellular Mtb-killing effect of PT-LE. In a similar manner, one of the natural products, bergenin-mediated suppression of Mtb survival was restored by inhibition of ERK activation within mouse peritoneal macrophages [30]. The present findings indicate that PT-LE potentiated Mtb-triggered immune signaling pathways, including the MAPK pathway, thus promoting bacterial clearance within macrophages.

Furthermore, it was hypothesized that post-treatment with PT-LE following Mtb infection would result in increased production of inflammatory cytokines when compared with Mtb infection alone. However, only MCP-1 production was significantly elevated by PT-LE during Mtb infection. The production of TNF-α and IL-6 was not elevated by PT-LE stimulation in Mtb-infected macrophages. Consequently, PT-LE appears to orchestrate a balanced immune response: it curtails the pathological release of tissue-damaging cytokines without compromising, and instead bolstering, targeted intracellular killing of Mtb. A recent study demonstrated that MCP-1 plays a direct role in promoting antimicrobial activity through NO production [31]. In our study, PT-LE also significantly increased NO production during Mtb infection. The present findings demonstrate that MCP-1-induced NO production by PT-LE functions as a significant host defense mechanism for bacterial elimination. Further studies are needed to establish the effects of PT-LE-induced inflammatory cytokines on Mtb infection.

Our results suggest that PT-LE treatment reduces the accumulation of p62 and LC3-II during Mtb infection, both *in vitro* and *in vivo*, indicating an enhancement in the maturation of autophagosomes into autolysosomes. Similarly, natural and synthetic agents including resveratrol and metformin have been demonstrated to eliminate intracellular Mtb by autophagy inducing autophagy through the upregulation of phagosome-lysosome fusion [32, 33]. In the present study, the resolution of autophagy flux obstruction due to Mtb infection by PT-LE treatment was observed. This finding suggests that the reduction in Mtb burden may have contributed to a decrease in inflammatory cytokine production, thereby mitigating lung tissue damage. Although the *in vivo* administration of PT-LE mitigated this lung immunopathology without inducing systemic body weight loss, a limitation of the present study is the lack of comprehensive biochemical toxicity profiling. Future preclinical evaluations incorporating the quantitative analysis of serum hepatic AST/ALT and renal markers are needed to definitively establish the safety and tolerability of PT-LE.

A notable benefit of HDT agents is their capacity to augment the effectiveness of existing antibiotics [34]. In the present study, the combination of PT-LE demonstrated a synergistic effect in reducing the Intracellular MICs of RIF, INH, and EMB against intracellular Mtb, with FIC values falling below 0.5. Consistent with our findings, a combination of some phytochemicals, including curcumin and bergenin, with INH has been shown to enhance the efficacy of INH in reducing intracellular survival of Mtb [35, 36]. While these synergistic effects were established *in vitro*, rigorous long-term *in vivo* studies using murine models are imperative to further validate the translational potential and therapeutic efficacy of PT-LE-based combination therapy.

In our study, we demonstrated that PT-LE exerts a potent HDT effect against Mtb infection by modulating the MAPK pathway and autophagy flux. The synergy between PT-LE and anti-TB drugs suggests that PT-LE could improve standard therapy as a potent HDT agent.

## Supplemental Materials

Supplementary data for this paper are available on-line only at http://jmb.or.kr.



## Figures and Tables

**Table 1:** Synergistic effect on intracellular MIC of PT-LE with anti-TB drugs against intracellular Mtb.

Species			Alone		Combine		
			Intracellular MIC (μg/ml)	Intracellular MIC (μg/ml)	FIC^*^	FIC index^**^
*M. tuberculosis* (H37Rv)	PT-LE + RIF	PT-LE	50	6.25	0.125	0.43 (Synergy)^***^
		RIF	0.1	0.03	0.3	
	PT-LE + INH	PT-LE	50	6.25	0.125	0.43 (Synergy)^***^
		INH	0.1	0.03	0.3	
	PT-LE + EMB	PT-LE	50	6.25	0.125	0.2 (Synergy)^***^
		EMB	4	0.3	0.075	

The intracellular MIC of PT-LE alone and in combination with RIF, INH or EMB against H37Rv were determined in infected macrophages. BMDMs were infected with H37Rv infection (MOI = 1) and then treated with PT-LE and anti-TB drugs for 24 h.

^*^ Fractional Inhibitory Concentration, FIC = MIC_combine_ / MIC_alone_

^**^ FIC index = FIC_PT-LE_ + FIC_anti-TB drugs_

^***^ Type of interaction (categorization via FICI values: synergy as FIC ≤ 0.5, indifference as 0.5 < FIC ≤ 4, antagonism as FIC > 4.

INH, isoniazid; RIF, rifampicin; EMB, ethambutol
