# Supplementary material for: Philadelphus tenuifolius Leaf Extract Exhibits Anti-Tuberculosis Activity by Enhancing Host Autophagy and Immunity: A Promising Host-Directed Therapeutic Candidate
Source: J Microbiol Biotechnol. 2026 Mar 26;36:e2601032. doi: 10.4014/jmb.2601.01032 (PMC13036502; doi:10.4014/jmb.2601.01032)
Supplement: Supplementary file 1 [file jmb-36-e2601032-supple.pdf]

J. Microbiol. Biotechnol. 2026. 36: e2601032

https://doi.org/10.4014/jmb.2601.01032

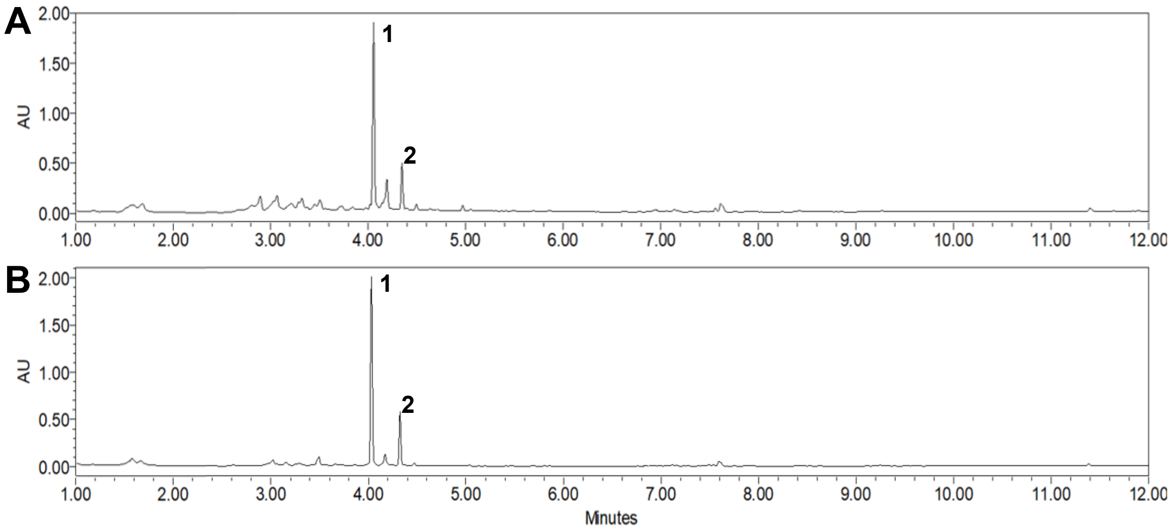

Fig. S1. HPLC chromatographic profiles of (A) *Philadelphus tenuifolius* leaf extract (PT-LE) used in the present study and (B) an authenticated standard extract obtained from the Plant Extract Bank at the Korea Research Institute of Bioscience and Biotechnology (KRIBB), Republic of Korea. Chromatograms were recorded at 254 nm. Two major peaks were consistently observed in both extracts and identified as (1) rutin and (2) nicotiflorin. These compounds were used as marker constituents to ensure chemical consistency and reproducibility of PT-LE.

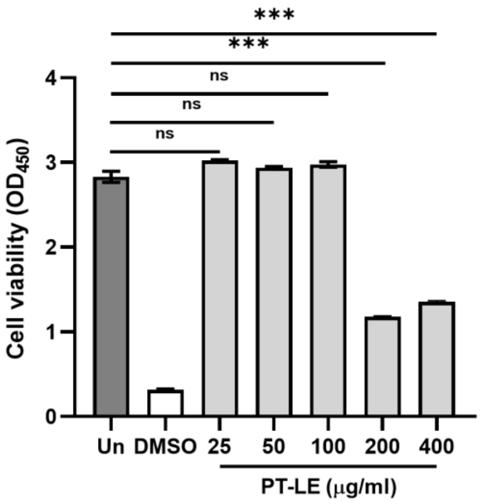

Fig. S2. Minimal cytotoxicity of PT-LE in BMDMs.

BMDMs were treated with increasing concentrations of PT-LE (25, 50, 100, 200 and 400 μg/mL) for 24 h. Cell viability was assessed using MTT assay and the value of optical density (OD) were measured at 450 nm. DMSO (10 %) was used as positive controls. Data represent mean ± SEM from three independent experiments. Un, untreated control. n.s, not significant.

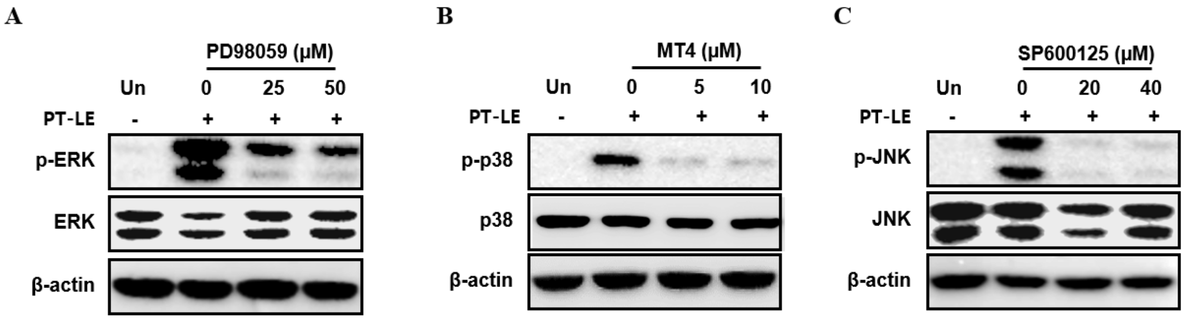

Fig. S3. MAPK inhibitors effects in BMDMs during PT-LE treatment.

BMDMs were pretreated with specific MAPK inhibitors, including PD98059 (25 and 50 μM) **(A)** MT4 (5 and 10 μM) **(B)** or SP600125 (20 and 40 μM) **(C)** for 1 hour followed by stimulation with PT-LE (100 μg/mL). Western blot analysis was performed to detect phosphorylated and total forms of ERK, p38, and JNK. β-actin was used as a loading control.

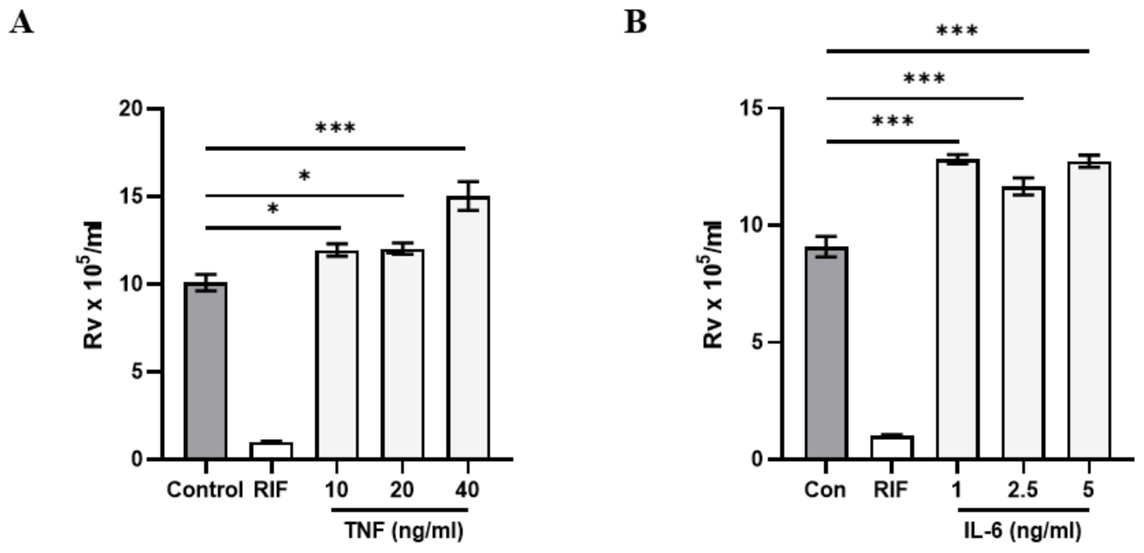

Fig. S4. Recombinant TNF-α and IL-6 show no effect in reducing intracellular survival of Mtb in BMDMs.

BMDMs were infected with Mtb (MOI = 1) and then treated with recombinant TNF-α (10, 20, or 40 ng/mL) **(A)** or recombinant IL-6 (1, 2.5, or 5 ng/mL) **(B)** for 24 h. Intracellular bacterial survival was determined by CFU assay. RIF was used as a positive control. Data are shown as mean ± SEM from at least three independent experiments. Statistical significance was determined by one-way ANOVA with Tukey's post-hoc test. *$p < 0.05$, ***$p < 0.001$. RIF, rifampicin.

Table S1. Limited direct bactericidal activity of PT-LE against Mtb.

Species	MIC/MBC PT-LE (µg/ml)
M. tuberculosis **(H37Rv)**	**>100/>100**

M. tuberculosis H37Rv was inoculated 1 x 10^5/mL in 200 mL 7H9 broth containing 10 % OADC with the presence of PT-LE. The MIC and MBC were determined after 21 days. MIC and MBC tests were performed at least twice.
